# A Course of Lectures on Dental Physiology and Surgery, Delivered at the Middlesex Hospital School

**Published:** 1848-01

**Authors:** John Tomes

**Affiliations:** Surgeon Dentist to the Hospital.


### ARTICLE II.

*A Course of Lectures on Dental Physiology and Surgery, delivered at the Middlesex Hospital School.* By JOHN TOMES, ESQ., Surgeon Dentist to the Hospital.

[Continued from page 54.]

### LECTURE VIII.—Continued.

IRREGULARITY IN THE POSITION OF THE WHOLE OF THE FRONT TEETH—IRREGULARITY IN THE CENTRAL INCISORES, IN THE LATERAL INCISORES, AND IN THE CANINE TEETH—IRREGULARITY IN THE BICUSPIDES AND MOLARES—IRREGULARITY IN THE POSITION OF THE WISDOM TEETH, AND THE CONSEQUENT DISEASES.

THERE is a fourth form of complete irregularity of the front teeth: it is this. The alveolar lines of the jaws are developed at such an angle with each other, that on closing the mouth the molar teeth alone come in contact, the front teeth being separated by a considerable interval. The models on the table present a well-marked case of this peculiar formation. If the defect in formation be discovered early in life, some attempt at restoration should be made by continued pressure under the chin; but if the deformity is found in the adult, the less you interfere the better, as your patient will have overcome, by habit, the inconveniences arising from malformation.

#### *Partial Irregularity of the Front Teeth.*

At present we have treated of irregularity affecting the whole of the front teeth; we shall now consider malposition when occurring to a part of the front teeth, and in doing so we will commence with the central incisores.

The central incisores, one or both, may project forward or retreat backward in an unnatural degree. Of these deformities the latter is the most common, and the one which we are the most frequently called upon to remedy. The cause which usually produces these misplacements is the prolonged presence of the temporary incisores, which oblige the new tooth to take either a posterior or anterior position, while the milk teeth is forced outwards or backwards, the proper position of the new tooth being between the two positions.

The central incisores may, however, occupy the proper line, but yet be irregular. The teeth may be twisted on their axis, either towards or from the mesial line; that is, the mesial edge may be turned inwards while the internal side projects forward in such a manner that the cutting edges of the two teeth form an angle with each other, the angle pointing towards the mouth; or the position may be reversed, and the angle point outwards.

This form of irregularity is, like the last, produced by the relative position of the temporary and permanent teeth during the development of the latter. Thus the mesial angle of the cutting edge is placed anterior to the fang of the central, while the external angle is placed behind the fang of the lateral temporary tooth; or the position may be reversed, the external angle may be anterior to the fang of the temporary lateral. In each case, unless the temporary teeth are shed at an early period, the permanent tooth will, on coming through, be at an angle with the proper dental curve.

The presence of the temporary lateral, when the centi al is shed, may also lead to malposition of the permanent central, supposing the central be a large tooth, and the space destined for it be too small.

The lateral incisores are subject to the same forms of irregularity as those which occur to the central teeth, and, therefore, do not require a separate description. It may be observed that the more common malposition is the direction inwards, and consequent closure behind the teeth of the under jaw on shutting the mouth.

The causes producing irregularity of the lateral are similar to those effecting malposition in the central teeth, namely, want of space for them to take the proper position; the prolonged presence of the temporary teeth, irregularity of position as regards the position of the fangs of the temporary teeth during development, or irregularity in the teeth of the under jaw, forcing the upper teeth on closing the mouth.

*The canine teeth of the upper jaw* may be turned sideways, may stand anterior to the adjoining teeth, which is the most common form of irregularity, or may incline inwards, which is the least common of the irregularities of position occurring to these teeth.

The circumstance of the late protrusion of the canine tooth in a small jaw is a constant source of irregularity; the incisores anteriorly, and the bicuspides and the first molar teeth, having taken their places posteriorly, insufficient space is left for the canines, and as they occupy when developing a rather anterior position in the jaw, they most commonly are evolved anterior to the neighboring teeth.

*Treatment.*—Whichever of the anterior teeth be the subject of malposition the same modes of treatment for the reduction of the irregularity are applicable. If, then, the front teeth are found, on examining the gums, to be out of position, the cause of the irregularity should be sought.

If there is a want of space, one or more of the temporary teeth should be removed, and the patient should be directed to press the new tooth frequently during the day towards the proper situation. The required position may, in some cases, where the irregularity is slight, be given to the new tooth by the frequent use of a piece of wood, shaped something like the handle of a spoon. The bent part should be passed behind the inward tooth, and the mouth partially closed, when the projecting portion of the instrument should be pressed downwards towards the chin. The under teeth will then form the fulcrum, and the upper tooth will be moved outwards. If the under teeth stand too much inwards the action of the lever may be reversed, and the upper teeth made the fulcrum. The teeth forming the fulcrum of the lever may also be moved, though probably to a much less extent than those acted upon by the end of the lever, as in the one case the pressure bears upon the concavity of the dental arch, and in the other upon the convexity.

If the teeth are sufficiently developed in length to pass behind the under teeth when the mouth is shut, these means will not be sufficient to restore them to a proper position, because the under teeth by their anterior position prevent them from coming forward. Our first step in the treatment must, therefore, be to remove the influence of the opposing teeth by placing caps upon the molars, which will so heighten them that on closing the mouth the front teeth do not come in contact.

Having accomplished this point, and ascertained that there is sufficient space in the proper situation, steady pressure must be made upon the irregular teeth. If one tooth only be displaced it may be sufficient to pass an elastic ligature behind or before (as the case may require) the irregular tooth, and then round the adjoining teeth, in such a manner that the ligature shall exercise a tractile force upon the tooth in the direction in which it should move. The best material for elastic ligatures is vulcanised caoutchouc.

If, however, the irregular teeth are well grown, the treatment by elastic ligature may occupy an inconvenient time, and it will be better at once to construct a plate fitting the palate, and thus gain a fixed point. If the teeth are in-placed, we may force them outwards by interposing pieces of compressed wood between the plate and the irregular tooth, or if the irregular tooth stand external to the proper line, an elastic ligature, or short bit of gold spiral spring, may be connected to the plate and to the tooth: the spring, by its action, will drag the tooth into its place. In most cases the tooth will move more quickly than the gum will absorb; it is necessary to bear this in mind, that sufficient space may be left for puckered gum.

A very simple and effective method of moving an upper tooth or teeth that are placed internal to the proper line consists in making an artificial elongation of the under teeth, the anterior surface of which inclining inward must pass, on closing the mouth, behind, and press outwards the in-placed teeth, while the base is fitted and firmly fixed to the under teeth. This instrument, which may be made of gold or of ivory, must be worn continuously by the patient till the irregular teeth have assumed the natural position.

If a tooth is twisted upon its axis, our treatment must be modified; pressure must then be made upon the side of the tooth which is most misplaced. But before proceeding to use mechanical means, observe whether the evil is kept up by the opposing teeth; and, on examination, you will frequently find that the edge, when the mouth is closed, rests between the two opposing teeth, one angle of the twisted tooth lying in front, and the other behind its opponents. In this case the molar teeth must be capped so as to remove the reciprocal action of the antagonist teeth. To one of the caps a piece of elastic gold wire may be firmly united; the free end must then be brought in front of the twisted tooth, upon the receding side of which it must be made to act. The better method of connecting the spring to the tooth is to fit with great accuracy a band of metal round the crown, to which band a small loop may be soldered; the end of the spring may then be drawn down and secured, by ligature to the loop. Or, instead of using the spring external to the teeth, we may attach a spiral gold spring to a fixed ivory palate, and to the tooth, through the medium of a band and loop, as in the former case, and thus turn the tooth on its axis till it assumes the proper line. I believe in a few instances the central or lateral incisores have been, by the use of the forceps, at once twisted into their proper position, and then secured by ligature to the adjoining teeth. I cannot recommend the practice, as it will, in many cases, be attended with the loss of the tooth.

It has been shown that pressure regularly applied upon a tooth in any other direction than in a line with its axis will gradually move the tooth in the direction of the pressure, whatever be the age of the patient: it follows, therefore, that dental irregularity may be reduced at any period of life. Whether it is advisable at all ages to reduce by mechanical means outstanding teeth is a question we must now consider.

If the patient be young, and the irregularity is not kept up by the closure of the teeth, it will be sufficient to remove the cause of the malposition by the extraction of the obstructing temporary teeth; or, if it be necessary, a permanent tooth.

The teeth will then during the general growth of the body, arrange themselves into the proper line. There is a strong tendency in the teeth to assume their proper relative position, and if the obstructive cause be removed sufficiently early, they will, though at first considerably out of place, gain of themselves the desirable position.

If, however, the irregularity is kept up by the closure of the antagonist teeth, the sooner after the full evolution of the tooth mechanical means are resorted to the better.

In the adult, if teeth are misplaced from want of room for a regular arrangement, one of the permanent teeth must be sacrificed; and it may, if the maxilla is very small, be necessary to remove a tooth from each side of the jaw. The tooth selected should be either the second bicuspid or the first molar; the reason for taking one or other of these teeth in preference to a more anterior tooth will be shown by a statistical table illustrative of the relative value in durability of each tooth. After making space by the removal of a tooth, the anterior teeth must be forced backwards by pressing between them small pieces of compressed wood, or India-rubber: the former by swelling, the latter by its elasticity, will force the teeth towards the vacated place. Having thus gained sufficient space, the irregular tooth must be brought into its place by the means I have before described; for we must recollect that in the adult the disposition in the teeth to assume the proper line no longer exists.

It is necessary to bear in mind, that although a tooth may be moved either from side to side, or backwards and forwards, yet that we have no power of lengthening a tooth. If, therefore, an irregular tooth be short, and its edge placed upon a different plane to the cutting edges of the adjoining teeth, and at the same time stands considerably out of line, it may be necessary to remove the tooth, even though it be a front one. For if you bring a tooth into the line, and when there it is shorter than its neighbors, it will still be unsightly. The canine tooth of the upper jaw is especially liable to come through the gum at a much higher level than the adjoining teeth, and if space be not afforded it to take its position the development of the tooth will be completed in the irregular position. The crown will be the usual size, but the fang will be very short. When these cases are met with the tooth should be extracted, for it is not only irregular in position but in conformation, the fang being unnaturally short. Before sacrificing the tooth, we should satisfy ourselves that its development is completed, for if the fang is not fully formed, the tooth may be brought into its place, and the development of the fang will proceed. You will see how desirable it is to preserve the front teeth, and especially the canine, when we come to the statistical tables of the relative durability of the teeth.

A tooth that has been moved by pressure feels to the patient, and, indeed, is during the process, and for a short time afterwards, slightly loose, and exceedingly sore. In the young subject and in the adult the tooth on being left at rest soon loses this tenderness and fastens; not so, however, in the decline of life. On the contrary, pressure upon a tooth induces irritation in the gum and alveolus; the latter becomes absorbed, and the tooth when left at rest seldom becomes as firmly placed as before the operation, neither is the absorbed alveolus reproduced. In order to prevent any injurious motion in a tooth, after being by mechanical means brought into place, a silk ligature should be passed round the neck of this and the adjoining teeth, and securely tied. The ligature, if efficiently applied, will not only prevent motion, but will also counteract any tendency in the tooth to recede to its former position.

*The bicuspid teeth are sometimes, from want of space misplaced;* the crown may project inwards towards the tongue, or outwards towards the cheek. If the degree of irregularity be slight, the teeth being but little seen, may be allowed to remain; if, however, any inconvenience be felt, the irregular tooth should be extracted. Where there is a choice the second should be removed in preference to the first bicuspid.

The first and second molares are so seldom the subjects of irregularity in position that we may pass them over.

At present I have been speaking more especially of malposition of teeth of the upper jaw; what has been said of abnormal position occurring of the one may, however, be applied to the other jaw. I need not, therefore, occupy our time with a description of the dental irregularities occurring in the lower jaw. The treatment will be similar in principle to that adopted for the upper teeth, though the apparatus must, of course, be modified to suit the form of the jaw. Thus, when we require a fixed point from which to act upon a lower tooth, it must be external to the teeth. The under teeth seldom require mechanical means to reduce them to regularity. In the first place they are in the majority of individuals but little seen; slight irregularity is, therefore, but little regarded. Then, again, the malformation is generally the result of want of space, and passes away on the removal of the crowding tooth. The tooth selected for removal should, in the majority of cases, be the first molar, from its great liability to early decay.

In describing to you the various forms of dental irregularity, and the treatment to be adopted, I have endeavored to lay before you the general principles, and especially as regards the treatment, in which I have described the plans usually adopted, in preference to giving an account of the construction of the instrument to be used in each case. I have done so, first, because we have not sufficient time allotted to us to go minutely into every part of the subject of dental surgery, and, secondly, because if I did, such information would be of but little value to those destined to practise exclusively medicine or surgery.

*Irregularity in the position of the wisdom teeth* demands our earnest attention, not so much on account of the disfigurement it may occasion, as on the serious and distressing disorders that are sometimes consequent upon misplacement.

The wisdom teeth of the upper jaw, when taking a false direction, give rise to great inconvenience, but the more distressing symptoms are generally connected with malposition in the inferior maxilla. I shall, therefore, direct your attention more especially to the latter, as in those cases we shall find the disorders similar, but more severe and better marked than when arising from the wisdom teeth of the upper jaw.

The third molar may be malplaced in five different directions.

First, it may grow obliquely forward with the masticating surface directed against the posterior surface of the second molar. Second, the crown may be directed outwards towards the cheek, and may even be imbedded in the cheek. Third, it may take an inward direction towards the tongue. Fourth, the tooth may be directed upwards in the coronoid process, either completely or partially within the bone. Fifth, it may occupy its natural position in the jaw, but be held down by indurated gum.

If we recall to our minds the progressive stages of the development of the three molares we shall be able to assign the causes of these various forms of irregularity. You will remember that, immediately after the development of the sac for the wisdom tooth, it ascends into the coronoid process, that the ramus of the jaw increases in length, and that when sufficient space is formed the sac descends and forms a line with the more anterior teeth; but if fro many cause the jaw ceases to elongate when the sac occupies the coronoid process, the tooth will be developed in that situation. On the other hand, if the maxillary growth stops when the sac has descended to the angle only, the tooth will be directed forwards. Again, if growth should cease when the tooth has descended and fallen into the line, but before sufficient space has been formed for its evolution in the vertical direction, the tooth will be directed outwards or inwards, taking that direction in which the least resistance is offered. It may happen, however, that although there is sufficient space, in the alveolar line, yet the wisdom tooth does not appear through the gum, arising, as far as we can tell, from the presence of a thickened and indurated gum, which offers effectual resistence to the eruption of the tooth.

Cases illustrative of the evils that occasionally arise from the various forms of malposition of the wise teeth not uncommonly present themselves to our notice. I shall, however, place before you several cases related by Velpeau; first, because they are very instructive in themselves, and secondly, because they come with the weight of his authority.

I should also, but for the want of time, cite some cases from Dr. Ashburner, to whom the profession is deeply indebted for bringing before their notice the various evils that may and sometimes do arise from malposition of the wisdom teeth.

Case 1.—"A lady, at the age of 22, began to feel a dull pain at the angle of the lower jaw on the left side of the face; the pain soon extended to the adjoining teeth, but was distinct from toothache. As the pain continued to increase in intensity for several months it was thought to be a case of rheumatism, and as such was treated, but without good effect; then blisters, and a seton at the back of the neck, kept open for a month: opiates were given, but all to no purpose. She went and resided at a watering place for some time, but came back to Paris nothing benefitted. At this time the teeth were all good in appearance, the gums healthy, and nothing denoting the eruption of a wise tooth. However, upon making a section of the gum over the wise tooth, a probe passed down led to the discovery that the wise tooth was arrested in its progress by the direction it had taken directly forwards, its crown coming in contact with the posterior surface of the second molar. The second molar was extracted, and the patient immediately released from her suffering."

Case 2.—"A lady, 29 years of age, sought advice on a painful tumor in the cheek: it had existed for several months. On examination, it was found to arise from the wise tooth projecting horizontally outwards, and lodged in the parietes of the cheek. So soon as the mouth could be opened sufficiently wide the tooth was extracted, and the patient soon recovered."

Case 3.—"A gentleman, of 45 years of age, had suffered from an ulcer on the side of the tongue, near its base. This ulcer he conceived to be syphilitic, and so was salivated as a means of cure. The salivation, however, made it much worse. After a while, on application to Velpeau, it was found that the wise tooth on that side was directed inwards, and projecting into the mouth had occasioned the ulcer on the tongue: the tooth was extracted, and in a few days the ulcer healed."

Case 4.—Patient F. Boulanger applied to Velpeau in 1825. The right cheek greatly swollen, the tumefaction extending from eyelids to the clavicle; several cicatrices, resulting from old abscesses, mark the skin in this neighborhood. For the last twenty months the patient has not been able to open his mouth, and has therefore subsisted on broths. Three inches down the neck from the angle of the jaw there is a fistulous opening, through which much pus is discharged; lower down the neck there is another fistulous opening. On passing a probe into the first of these openings it penetrated obliquely backwards about three inches, and then was stopped by a hard body, which proved to be the dens sapientia. The patient's health had suffered much from frequent attacks of colic and diarrhœa, indigestion, &c. The mouth was gradually forced open by the introduction of bits of cork and wood between the teeth; the process was tedious but successful. The tooth was extracted, and in five or six days a sequestrum was discharged which seemed to belong to the coronoid process; it bore the mark of the crown of the tooth, and thus indicated the obstacle which had opposed the development of the tooth. The second molar was now extracted: eight days afterwards a second piece of bone was removed; and from this time the tumefaction of the neck and face disappeared so rapidly that at the end of twenty days the face had resumed its natural appearance.

CASE 5.—A gentleman of fifty years of age had, during the last two years, suffered excessive torture. The right side of the face was greatly swollen and disfigured by sores from old abscesses; the neck, also, was also swollen down to the clavicle, and marked in a similar manner; the mouth remained half open, and was distorted, the lower teeth not corresponding in range with the upper. The general health of the patient had been considerably affected by his sufferings. For the last four months he labored under constant diarrhœa; fetid saliva, mixed with pus, flowed constantly from his mouth. He was feeble, emaciated, and unable to walk without the support of two friends.

A mass of fungous flesh, which occupied the whole of the mouth on the diseased side, was freely divided, and, after a long examination, a wise tooth was found in the base of the coronoid process. The tooth and the whole of the process were removed, as were also several portions of the maxillary bone, with loose teeth. Within a fortnight all the bad symptoms had disappeared, and nothing remained but the deviation of the mouth, which was finally overcome by means of a bandage.

CASE 6.—Dr. Fricard, when a student, was attacked, in the summer of 1821, with pain in the throat, and in the following November with severe inflammation of the right tonsil. This condition was partly subdued by antiphlogistic measures; but the pain soon returned, and continued, in spite of every means, up to the year 1823. The teeth and gums appeared to be perfectly healthy, and the surgeon was about to extirpate the tonsil, when it was accidentally discovered that the wise tooth on the affected side was not through. The gum was now freely divided, but the portions of the divided gum inflamed, and had to be removed with the knife and caustic. The tooth was thus completely freed, and the obstinate inflammation of the tonsil soon disappeared.

CASE 7.—M. Esquirol informed Velpeau that he had a case of mental derangement where the patient was restored to reason by a crucial incision division of the gum, which liberated the wise tooth.

These cases were related by M. Velpeau in a clinical lecture which was reported in the Medical and Surgical Journal in 1841.

We should at first sight scarcely expect that consequences so serious would arise from the development of the wisdom teeth, when occupying an abnormal position; neither do they necessarily occur when the teeth occupy either of the situations I have described. Before serious and painful results can arise there must be a concurrence of many circumstances. The patient must be of strumous constitution or predisposed to inflammatory action, or the nervous system must be peculiarly susceptible of excitement; in which case epileptic fits may supervene upon any interruption to the advance of the wisdom teeth. That epilepsy does arise in some cases from this cause is proved by its appearing when the teeth are passing towards the surface, and disappearing when passage is given to the teeth by removing the obstructing cause. I have, within a few weeks of this time, known of three cases which have occurred in the patients of medical friends, where fits have commenced when wisdom teeth were presenting, and which were completely removed by free incision through the indurated gum over the teeth. As a precautionary measure, lint should be placed in the incisions to prevent the union of the divided surfaces, otherwise the symptoms may recur.

I have during the last two years seen four cases in which the teeth presented with the crown forward, and produced great pain about the angle of the jaw, extending to the neck and shoulder or to the side of the head. In each case the wisdom teeth were removed. The operations were attended with considerable pain, but it is better that pain should be suffered than that the second molar should be lost in order to give space for a tooth which, when fully developed, will be useless in mastication.

The wisdom teeth when malplaced are wholly useless in mastication; we need not, therefore, hesitate to remove them whenever they give inconvenience. These teeth, although in their proper situation in the jaw, yet may, from their closeness to the ascending ramus, give great inconvenience, not from want of space for the tooth itself, but from the mucous membrane of the ramus lying over the posterior surface of the teeth, and being, consequently, subject to injury from the upper teeth whenever the mouth is closed. Inflammation is thus produced, which in a susceptible subject will not only be kept up in the injured tissues, but will extend to the throat and tonsils, and then remain in a chronic state so long as the cause is allowed to exist.

In these cases the better remedy is to remove the tooth, seeing that, from its position, it is wholly useless; or, if this be objected to, the overlapping piece of membrane should be removed with a pair of curved scissors; a remedy may also be effected by the frequent application of caustic.

It is obvious that the evils produced by the development of irregularly placed wisdom teeth arise wholly from pressure produced by the gradual growth of the dental pulp, preparatory to its calcification; by which the superimposed tooth is forced against the tissues by which it is covered. In the natural course of things these tissues are absorbed, and make way for the rising tooth; but if they are not absorbed, and, therefore, resist the advance of the tooth, the pressure reacts through the tooth upon the developing pulp.

The pulp is very plentifully supplied with nerves, and, moreover, is placed immediately over the large dental nerve (at least in the under jaw.)

We all know that pressure upon a nerve or nerves, though slight in degree, will produce more or less pain, and that the pain will be felt in the part to which fibres pressed upon are ultimately distributed. Instead, then, of being surprised that interruption to the growth of a tooth should produce pain extending through the whole jaw, or to the side of the head, or about the ear, we should be led to infer that such phenomena would exist. It would be, perhaps, difficult to estimate the actual amount of mechanical force developed by a growing tooth, but we may form some estimate by observing the force generated by growing vegetable. We have most of us seen a tolerably weighty piece of stone raised by the growth of a plant beneath, any fibre of which may be readily crushed beneath the thumb and finger.

Pressure, moderate in degree and equally applied, will lead to the absorption of tegumentary tissues; but if the pressure be great, and unequally applied, instead of absorption we have induration and pain—a fact those who have worn tight boots are willing to admit. If a tooth be developed more rapidly than the superimposed gum is absorbed, gradually increasing pressure will be applied to the gum, which will then become indurated and painful, and the symptomatic disorder I have alluded to may arrive.

In all cases of inconvenience arising from malposition of the wisdom tooth, either the tooth itself or the parts obstructing its development must be removed.

The evil will, however, if not in itself considerable, and the patient has sufficient endurance, after a time terminate. The development of the tooth will cease in the formation of very short fangs, and the pressure no longer generated, will cease, and the patient will be restored to comfort.

I have omitted to mention, that irregularity in position is sometimes caused by tumors growing from the gums or alveoli, but as we shall, in a future lecture, consider the history of diseases of that nature, I need not detain you now by describing their effects.

### LECTURE IX.

OSSEOUS UNION OF TEETH TO EACH OTHER—UNION OF TEETH TO THE ALVEOLI—MECHANICAL INJURIES—FRACTURE OF TEETH—FRACTURE EXTERNAL TO THE PULP CAVITY—THROUGH THE PULP CAVITY—THROUGH THE FANGS—UNION OF FRACTURED TEETH—DILACERATION OF TEETH—INJURIES OF THE PULP—DISLOCATION OF TEETH—FRACTURE OF THE ALVEOLI.

*Irregularity in the Structural Relations of the Teeth with the Adjoining Tissues.*—It is by no means uncommon to find the fangs of a molar tooth, which are usually separate, united; but further there are many well authenticated cases where the fangs of two adjoining teeth have been permanently connected. A case occurred in the practice of my friend Mr. Rogers, in which the second and third molares of the upper jaw were firmly united. If my memory does not fail me, the wisdom tooth was decayed and painful. On attempting to remove the offender, both teeth came out, and it was then found that they were united by their fangs. It is probable that junction was effected between the bone or cementum of the fangs. Although such cases are rare, yet there are a sufficient number recorded to establish the fact. Mr. Bell describes several cases from preparations in his own possession. In the American Journal of Dental Surgery, an instance is mentioned in which the lower bicuspides were joined.

Before we leave the subject of dental union, I shall take the opportunity of bringing before your notice a very interesting specimen which I had the good fortune to procure from an ivory dealer.

The tusk of the elephant grows during the whole life of the animal; the older the elephant, the larger the tusk. Usually there is a single tusk developed from each side of the upper jaw. In the specimen before you, three tusks have grown from one side—a large one above, and two smaller ones immediately below. But the great peculiarity exists in this, that the apices of the smaller ones are united to the under surface of the larger one. The union is affected not by the dentine of the three, but solely by the cementum. Each tooth has its separate alveolus and pulp cavity. Portions of the alveoli are still adhering to the surface of the lesser tusks.

*Osseous union between the Teeth and Alveoli.*—The fact as to whether the fangs of teeth are ever connected to the alveoli by osseous union, is not at present established. I have seen a specimen of a milk-tooth which remained in the adult jaw, and seemed to be united to the bone, but as a section of the parts had not been examined by the microscope the fact could not be considered as established. There seems no reason why union should not, under some circumstances, take place, yet, if such does occur, the cases are very rare, and are, therefore, practically unimportant.

*Mechanical Injuries of the Teeth—Fracture.*—The teeth, as well from their peculiar function as their exposed situation, are very liable to be fractured. The fracture may be external to the pulp cavity, in which case it may be termed simple, or it may extend through the pulp cavity, and thus constitute compound fracture. Compound fracture may be confined to the tooth, or may be accompanied by fracture of the alveolus.

*Fracture of teeth external to the pulp cavity, or simple fracture*, may be produced by the violent closure of the antagonist teeth, by biting any hard substance, or by a blow. If the dentine be exposed, pain will for a time be felt on subjecting the tooth to the contact of cold fluids, or even a current of cold air. A peculiar and destressing sensation is produced by any foreign body coming in contact with the exposed dentine. If left to take its course the pain and sensitiveness may wholly cease, or, on the other hand, the pulp may become inflamed, and the tooth consequently lost.

*Treatment.*—If the fractured edge of the enamel be rough, or unpleasantly sharp, a fine file should be used. The sensitiveness of the dentine may be destroyed by the application of an escharotic; nitrate of silver, potassæ fusas, or chloride of zinc in the state of powder, may be rubbed over the surface with a piece of wood rendered rough at the extremity, so that it will take up a little of the remedial agent: no doubt, there are many other substances which would answer the purpose. Those I have named will, however, prove efficient.

*Fracture through the pulp cavity* is of very frequent occurrence, and is usually the result of a blow or a fall. It may possibly be produced by the violent action of antagonist teeth when the muscles of the jaw are affected by spasm; though such a case has not come under my own immediate notice. The fracture may be situated in the crown, or in that part of the tooth which is enclosed within the alveolus, but as the two cases may lead to somewhat different results we will consider them apart.

When the injury has been confined to the crown, the fractured portion will of course be detached, and leave the pulp and surface of the dentine exposed to external influences. After an accident of this nature you will find the pulp projecting from the fractured surface, and extremely sensitive to the slightest touch, even of the tongue. Inflammation will shortly commence in the exposed pulp, from which it will extend to that portion situated in the fang, and from thence proceeded to the periosteum of the fang and alveolus. The inflammatory action may not stop here, but may extend further, and involve the periosteum of both the external and internal plates of the alveoli, and also involve the gum. In this case you will find considerable swelling of the face, diffused pain over the parts involved, accompanied with the constitutional signs of inflammation.

If allowed to take its course, the inflammation will terminate in the death of the pulp, and in the formation of pus in the alveolus, which will find its way down the side of the tooth, and be discharged under the edge of the gum, or a canal will be formed by absorption through the alveolus and the gum, and the matter will issue through the opening, which is commonly situated opposite the apex of the fang. Free exit being given to the pus, the inflammation will now abate, and the pain and swelling will subside with the escape of the matter. The tooth will become gradually dark in color. The secretion of pus will continue, though the quantity discharged may be so slight as to escape notice.

These are the consequences which, if no preventive means be used, usually follow upon a tooth being fractured through the pulp, and the pulp exposed but not destroyed. But if the accident occur in a strumous constitution the results may be more serious; the neighboring teeth may become involved in the inflammation, and the alveoli suffer necrosis; so that instead of the injured tooth alone being lost, the adjoining teeth, with the enclosing alveoli, may be destroyed.

The pulp may, however, be destroyed by the force which occasioned the fracture of the tooth; thus if the crown be broken off near the neck the pulp will probably be torn out of the fang by the detachment of the crown. On the other hand, the tooth may have been so moved in its socket that the vessels and nerves are severed at their entrance into the fang. In either case there is much less risk of inflammation than where the pulp is exposed to the immediate action of air, and the various substances taken into the mouth. The exposed surface of the dentine is in most cases for a while very sensitive, but the sensitiveness gradually subsides: the tooth becomes discolored, the discoloration commencing around the vacant pulp cavity, and extending outwards.

In a susceptible subject, however, you may have considerable inflammatory action following much the same course as occurs in those cases where the pulp is exposed but not destroyed.

In the most favorable case there is an attempt at reparative action. If the pulp be torn across half way down the fang, the remaining portion of the pulp will in the course of a little time seal up the exposed aperture of the cavity of the fang with newly-formed dentine, just as we see the cavity obliterated by new dentine in the fangs of teeth which have been much worn by mastication. In the one case the injured pulp withdraws itself from external influence by forming a layer of dentine on its exposed surface, and in the other by adding new dentine to the inner surface of the pulp cavity as the external walls are thinned by wear.

*Treatment.*—If the fang of a front tooth be sound, and the adjacent parts free from inflammation, a new crown may be pivoted upon it in such a manner that the artifice cannot be detected unless very closely examined, and the crown will answer all the purposes of the original one. (The manner of performing the operation I shall describe to you in a future lecture.) Hence, in many cases it will be desirable to preserve the roots of a tooth from which the crown has been broken. To effect this we must if the pulp be exposed immediately destroy it by thrusting into the cavity a small steel instrument; and when it can pass no further, from the narrowing of the canal as it approaches the end of the fangs, roll it between the finger and thumb; this will completely destroy the pulp soon after its entrance into the tooth; the coagulum formed from the torn and bleeding vessels will protect that little which remains. Having destroyed the pulp we may proceed to fix on by means of a pivot the new crown. Some dentists use platinum, or steel-wire, heated red hot, for destroying the pulp, under a belief that when the actual cautery is used the patient is less liable to an attack of inflammation of the alveolar periosteum as a result of the operation.

Four years since a gentleman had the central incisores broken off by a blow from a ball. I saw him on the same day. The pulp in each tooth was destroyed for some distance down the fang. The stumps were pivoted. No inconvenience followed the operation, and at the expiration of three years the roots of the pivoted teeth seemed perfectly free from disease.

Such should be our treatment if the fang be firm in the alveolus, and the patient applies to you before inflammation has commenced; but if the root be loose, from the blow which fractured the crown, or if the indications of inflammation are present, you should at once extract the root. If there is considerable inflammation about the alveolus, the patient should, after the removal of the tooth, be directed to foment the part freely, by taking into the mouth a strong decoction of poppy-heads, and at the same time an aperient should be given to reduce constitutional excitement.

If the accident has occurred at some distant period, and the now empty pulp cavity has its walls discolored, it will be inexpedient to pivot on a new crown, as in all probability the cavity gives passage to some slight discharge, which in being blocked in by the pivot will lead to abscess in the alveolus, for the relief of which the root must be extracted. You will in some cases find that pus, instead of passing out through the cavity, makes its escape through a small fistulous opening opposite the end of the root. In such a case pivoting may not make the matter worse, but as disease about the fang of one tooth is apt to extend to the adjoining tooth, it is far better to remove the root.

*Fracture of the Root.*—The root of a tooth, you are aware, is invested with periosteum, and fracture may occur without that membrane being detached, except at the line where the fracture has taken place. The tooth may, therefore, be retained in its place. If, however, the force causing the injury has been great, the fractured part may have been knocked out. The alveolus, by the same blow, may have been fractured, in which case there may be great displacement of the tooth, and in some instances without detachment from its socket. The direction of the fracture may be either transverse or oblique, and may itself be either simple or comminuted. The pulp, if the force causing fracture has not produced much displacement, may be but slightly injured, and vitality therefore preserved throughout its whole length;. on the other hand, if the displacement has been considerable, it must be torn across, and that part external to the seat of fracture destroyed.

You will recognise fracture of the fang of a tooth by the crepitus that will be felt on moving the crown. The fractured tooth, if left to itself, will, in many cases, produce inflammation and pain in the alveolus, which may oblige the removal of at least the fractured portion. This will not, however, be the necessary result; the fracture may unite.

It has been believed that a union of a fractured tooth could never occur, but the preparation which I am enabled to show you, through the kindness of my friend, Mr. Saunders, whose property it is, shows that union not only can, but in this instance has, taken place. The tooth is a central incisor of the upper jaw; the fracture extended obliquely through the middle of the fang, and was obviously attended with slight displacement, in which position perfect union has taken place. Several small nodules of dentine mark the line of union, the oblique direction of which, together with the displacement, are so well marked that there can be no doubt of the producing cause of the present appearance of the tooth—namely, fracture and subsequent union.

A second case of union is recorded in the American Journal of Dental Science, where you will see a wood-cut representing an incisor fractured near the neck, with the crown bent at a right angle with the fang, and in that position united.

It will not be difficult to understand how a tooth fractured through the fang may be united, supposing the pulp be not destroyed by the injury of the dentine. You have seen that when the surface of a tooth is worn, the pulp forms dentine in the cavity opposite to the worn external surface, and I shall show you that when a tooth becomes decayed, the pulp will, in some cases, form dentine in the pulp cavity, opposite to the seat of the disease situated on the external surface. The process of development in the two cases is similar to that engaged in the formation of the dentine of the body of the tooth. In each case we recognise reparative attempts, and also an unquestionable proof that where there is dental pulp, there we may have, under favorable circumstances, dentine developed. If, then, a tooth be fractured through the fang, and the pulp be not destroyed, a process may be set up similar in every respect to that effecting the union of fractured bones.

If we examine osseous union we shall be able to trace the following steps up to the completion of the process. Thus, the immediate consequence of fracture of a bone is the effusion of blood from the lacerated vessel of the injured part; this is followed by effusion of liquor sanguinis or lymph; the formation of cartilage between the fractured extremities then succeeds, and after a while the cartilage is converted into bone, and the union is complete. Similar action would no doubt arise when dental union takes place, but instead of cartilage we should have dental pulp, which would be converted into dentine. Numerous specimens of hypertrophy of the cementum attest that whenever there is dental periosteum there may be formed dental bone or cementum. The bony layer of the fang may, therefore, under favorable circumstances, be united.

*Treatment.*—If the fracture be situated about the middle of the fang, or near the neck, and the body of the tooth be knocked out, the remainder of the root, if painful, should be extracted; but if a small portion of the fang only remain in the alveolus, it is not well, unless it be painful or loose, to attempt the removal, as the operation would be attended with considerable pain. A small piece of root very seldom gives any inconvenience, and is, after a time, by the deposition of bone in the alveolus, brought towards the surface and thrown off, or it may be impacted in bone, and there remain without giving any evidence of its existence. Such has evidently been the case in the specimen before you; the bone is here closely fitted to the surface, even to a somewhat irregular fractured surface. Had there been any inflammation excited by the presence of the fang, we should see evidence of its existence in the adjacent bone. Instead of that, however, the texture of the bone immediately in contact with the root is free from unusual porosity common to inflamed bone, and is exactly similar to other parts of the maxilla.

Again a small portion of the fang of a fractured tooth may wholly or in part become absorbed. It must not be denied, however, that, in a few cases, the remaining portion of the fang of a fractured tooth will produce inflammation, and subsequently a gum-boil, which, had the stump been removed, would probably have been avoided. If after fracture the crown be not detached, but yet feels very loose in the gums, it should be removed; or if the gum be inflamed, it should be removed whether loose or not. Should, however, the fractured tooth be tolerably free from pain, and the gum from inflammation, and at the same time be held tolerably well in its place, we may attempt to get an union of the fractured surfaces. If we could insure perfect absence of motion in the tooth, it seems probable, judging from the specimen before us, that union might be effected. It will, however, be extremely difficult to insure perfect rest in the parts, and we know that even a bone will not unite unless there is perfect absence of motion between the fractured extremities, and much less would a tooth, when the nature of the new tissue, namely dental pulp, is much less firm, and therefore more readily injured than cartilage. From these circumstances our chances of success will, I fear, be small, though I think the chance worth the trial.

If several teeth with their alveoli are fractured, and there is displacement, the whole should be brought back to the proper position, and the patient should be enjoined to avoid all causes of motion in the injured part, which should be fomented, by taking into the mouth hot poppy-head fomentation, which should be renewed as soon as it cools. The general health should also be attended to, and aperients given if necessary.

*Dilaceration of Partially Developed Teeth from the Formative Pulp.*—By this I mean the forcible separation of the cap of developed dentine from the pulp, in which development of dentine is still progressing. I have but little experience in accidents of this nature. Dilaceration is of course the result of mechanical violence. Two cases have come under my notice, in which after severe injury to the jaw—in one from the kick of a horse, in the other from a fall—partially formed teeth came away, with necrosed bone. I am, however, through the kindness of my friend, Mr. Saunders, enabled to show you a specimen, in which there has been perfect reunion after dilaceration, and the development of the tooth has been perfected.

In this specimen the accident has evidently occurred subsequently to the development of the enamel, and there are also indications that there was slight displacement consequent upon the injury.

There is a distinct line of demarcation between the dentine formed before and subsequent to the accident; indeed the great distinctness of the line throws some doubt as to whether there is perfect reunion in this tissue. The dentine developed immediately after, and by which the junction or reunion is effected, is perforated by numerous canals, which were probably occupied by vessels, but which, like the vascular canals in the antler of the stag, become partially obliterated on the organ reaching maturity. The thickening or ridge on the external surface of the tooth consists of cementum, in which the reunion is very perfect, and is, I believe, perforated by vascular canals. The pulp cavity, you will perceive, is situated low down in the fang of the tooth, and at the upper part is studded with two small nodules of dentine. I have not seen a specimen which could be viewed microscopically by transmitted light, so that I cannot describe the structure more minutely.

The subject of reunion after dilaceration, though interesting physiologically, is practically of little value. The accident rarely occurs, and then is either not recognised, or if recognised is involved with such extensive injury of the adjoining parts, that there are no hopes of its coming into operation.

Mechanical injuries of the formative pulp, with partial dilaceration, though unknown in the human teeth, are occasionally met with in the tusks of the elephant, or perhaps I should speak more correctly if I say the results of such injuries are occasionally recognized in the tusks. Most museums possess specimens of tusk, in which balls are impacted, and everywhere surrounded with dentine. On close examination, it will, as far as my experience goes, be found that the dentine near the surface of the ball is slightly peculiar; that it has a translucent horny look, and in some specimens has a laminated appearance.

Before the development of dentine was understood it was extremely difficult to explain how ball should get into the solid part of a tooth. We can now, however, see that if a solid substance be lodged in the pulp, and remains there without producing inflammation, that it must, on the pulp becoming dentine or cement, be enclosed in the solid substance of the tooth, just as it was before enclosed in the pulp. But if the pulp near the foreign body be destroyed, then dentine is deposited at a short distance in such a manner as to enclose the ball loosely in a case, and at the same time protect the remainder of the pulp. The specimen on the table illustrates this point. The missile has evidently entered through the base of the tusk, and passed into the enclosed pulp. The fracture is unrepaired, but a loose case is formed round the ball, with an opening kept from without. This, which shows the marks of the fracture in their outline, has all the edges of the aperture rounded.

*Dislocation of Teeth.*—Dislocation may be partial or complete; that is, the tooth may be loosened considerably, and hang in its place, or it may be so completely detached from its articulation that it falls from the socket. Further, it may be complicated with fracture of the alveolus, or, on the other hand, the tooth may be driven up into the nasal cavity. Mr. Bell cites a case of this kind, which came under his immediate notice. Dislocation is always produced by mechanical violence when occurring suddenly, or by the growth of a tumor, or by deposition of bone in the socket, when occurring gradually. A front tooth is occasionally seen lengthened beyond its fellows; this arises from the gradual filling up of the alveolus from the bottom by the deposition of bone; hence the tooth is forced out, and appears to have lengthened.

If the dislocation be incomplete, and there be no injury to the jaw, the tooth will, if kept quiet, in a patient having a good constitution, become firmly refixed; but, on the contrary, we may have inflammation set up which will extend to the neighboring parts, and produce great mischief.

If the dislocation be complete, with but little injury to the alveolus, and the tooth has fallen out, the jaw usually gives but little trouble. It is common for a patient after the skilful extraction of a tooth from an uninflamed jaw to feel no subsequent pain whatever. If the dislocated tooth be driven into the jaw, great pain and active inflammation of the injured part may occur in any case, and will almost surely, if the tooth be not removed.

*Treatment.*—If a sound valuable tooth be completely dislocated, the alveolus should be immediately cleared of coagulum by the use of a strip of lint and a probe. The tooth should then be returned to its place, and fixed so as to prevent motion, by a silk ligature round the adjoining teeth. The patient should be cautioned against disturbing it by any attempt at mastication on that side of the mouth. By these means a dislocated tooth will often become firmly replaced in the jaw, though occasionally you will have inflammation arise as a consequence of the attempt. In all cases there will be some tenderness, and the tooth will for a short time be elongated and tender; a circumstance arising from swelling of the alveolar periosteum, and consequently slight displacement of the tooth. If, however, the tenderness be great, and accompanied by constant pain, all attempts to induce reunion should be abandoned.

Supposing a tooth to have become quite firm after dislocation, it does not follow that it will continue serviceable for any great length of time. Usually such teeth become discolored, being partially dead; from the connection with the vascular system existing only through the dental periosteum, and not through the pulp. The consequence is, that gum-boils not unfrequently appear, and these, if severe, may oblige the removal of the tooth; still, I have seen several cases in which teeth removed by mistake were returned to their alveoli united, and were useful for years.

If the dislocation be partial only, the tooth should be pressed back into its proper position, and in most cases it will unite to the alveolar periosteum. There is frequently a little difficulty in returning the tooth when the alveolus has become occupied by a coagulum. Firm pressure should be used, and when in place the tooth should be tied to the neighboring teeth.

You will have frequent failure, but yet it is always worth while to attempt to save a sound tooth, even if the chances of success are small.

If the dislocated tooth be driven into the antrum, or into the jaw, it should be immediately removed, otherwise inflammation will arise, which may end in necrosis of the injured bone.

*Mechanical Injuries of the Alveoli.*—Fracture arises from the same cause, and is usually connected with, fracture or dislocation of teeth. In some cases it is the result of unnecessary force, or force injudiciously applied in the extracting teeth. The extent of fracture will be in proportion to the degree of mechanical violence producing it, and may be confined to a simple fissure, or one or more pieces may be broken off. Fracture of the alveoli is necessarily concomitant with fracture of the jaw; but as this forms a subject in the surgical lecture we shall not touch upon it here.

It rarely happens that slight fracture of the alveoli in a perfectly healthy person is attended with serious inconvenience any further than some slight pain for a few days. If, however, your patient be in an unfavorable state of health, then necrosis of at least the injured portion, if not more, of the alveoli may be feared.

*Treatment.*—No doubt slight fracture is a very common result of the extraction of the teeth, as well as of other injuries of the teeth; but when the fracture is slight, and is, in fact, nothing more than a fissure, with but little separation, we have no means of ascertaining its existence, neither would the knowledge, could we obtain it, be of any practical value. Should, however, you detect a piece of alveolus broken from its connection with the maxilla, and attached only to the gum, it had better be removed, otherwise it will occasion irritation until it is thrown off by natural efforts.

Two cases have come under my notice where large portions of the alveoli have been torn away with the teeth, and in each the exposed surface of bone exfoliated. In the first case the two bicuspides and canine were removed with the enclosing alveoli in an effort to remove the second bicuspid with the key.

In the second case two molares, with their alveoli, were removed in the same manner, and the mucous membrane of the palate torn across as far as the soft palate. The patients applied to the hospital in consequence of the injuries received in the operations.

It is very common for a small bit of the edge of the alveolus to be found adhering to the neck of the tooth after removal. This amount of injury is very seldom of any account, neither need any treatment be adopted.

I need not detain you with any account of necrosis, as you will hear from Mr. Arnot or Mr. Shaw a far better description of that disease, whether situated in the jaw or elsewhere, than I should be able to furnish.

